# Beliefs About Cannabis Use Among Male and Female Andalusian Adolescents

**DOI:** 10.3389/ijph.2024.1606911

**Published:** 2024-05-31

**Authors:** María-Carmen Torrejón-Guirado, Shahab Jolani, Hein De Vries, Liesbeth Mercken, Marta Lima-Serrano

**Affiliations:** ^1^ Department of Nursing, School of Nursing, Physiotherapy, and Podiatry, Institute of Biomedicine of Seville (IBiS), University of Seville, Seville, Spain; ^2^ Institute of Care and Public Health Research, Faculty of Health, Medicine and Life Sciences, Maastricht University, Maastricht, Netherlands; ^3^ Department of Health Psychology, Faculty of Psychology, Open University of the Netherlands, Heerlen, Netherlands

**Keywords:** cannabis, gender, beliefs, subjective norms, adolescents

## Abstract

**Objectives:** This study assessed potential differences between girls and boys in the prevalence rates of cannabis use, sociodemographic factors, and beliefs about cannabis use.

**Methods:** 1,896 Andalusian adolescents aged 14–18 participated in an online survey based on the I-Change model. The survey assessed their beliefs about cannabis use, including attitudes, social influences, self-efficacy, action planning, and intention to use. Multivariate analyses of variance were then conducted to examine potential gender differences in these beliefs, while controlling for last month’s cannabis use.

**Results:** Significantly more boys used cannabis in the last month, had boyfriends/girlfriends, and had more pocket money compared to girls. Additionally, girls – in comparison to boys - were more convinced of the disadvantages of cannabis use, but were also more convinced of some of the advantages (such as freedom from boredom, and medicinal use), reported having less favorable social norms for cannabis use, had more female best friends using cannabis, and felt pressure to use cannabis from their female peers.

**Conclusion:** These findings highlight the need for cannabis prevention programs to consider gender differences in beliefs about cannabis use. Programs should not only address general risk factors for cannabis use but also evaluate if their interventions effectively target beliefs that are particularly important for girls and boys.

## Introduction

Cannabis use in adolescents is a serious public health problem throughout the world [1]. Differences between girls and boys may exist in terms of consumption patterns, beliefs, and social influences associated with this consumption [2]. The sex-gender system is a complex and crucial concept in health, social, and psychological analyses [3], and analysing sex differences on beliefs, including subjective norms, could help to understand this system, as beliefs can be shaped by the different social attributes of being a male or a female [4–6]. For example, an individual with biological male characteristics (i.e., sex) may be at a higher risk of adopting risky behaviours such as cannabis consumption (and therefore, having beliefs in favour of cannabis consumption), because it is what society expects of him (i.e., gender) [7]. So, research focusing on beliefs could address both concepts, sex, and gender (i.e., as social expectations, regarding how women and men should behave, are influenced by the biological sex, and can shape girls’ and boys’ beliefs about themselves and others, and their differential behaviour). Research assessing potential differences between girls and boys about beliefs about cannabis use is needed to examine whether gender-specific approaches to cannabis prevention are warranted [8, 9].

The adoption of risk behaviours, including cannabis use [10], is determined by different factors. The Integrated Change Model acknowledges the existence of a variety of different factors (such as predisposing and motivational factors that influence a person’s intention and health behaviour such as cannabis use [11]. Concerning the motivational factors the I-Change model assesses attitudes by assessing both rational and emotional advantages and disadvantages of the behaviour [12]. Concerning social influence beliefs, the I-Change Model distinguishes social norms (beliefs of others about the behaviour), social modelling (the actual behaviour that others are doing), and social perception of others carrying out this type of behaviour), and social pressure (the direct pressure to engage in risky behaviour exerted by others) [11]. Self-efficacy constitutes the third factor explaining a person’s intention and behaviour [13]. Additionally, pre-motivational factors, and post-motivational factors have also been acknowledged to facilitate further understanding of health behaviours as well as the importance of predisposing factors such as the socio-economical context [14]. Post-motivational action plans, such as plans to cope with challenges situations, are important to better understand the transition from intentions to behaviour [15], although research on this factor for understanding cannabis use is still scarce [16] and thus deserves more attention.

Previous international literature (mostly qualitative) identified certain beliefs, and social norms regarding the access and use of cannabis in boys and girls [2, 17, 18]. For instance, both, girls and boys, have been found to believe that cannabis use may facilitate women’s access to the male world/the society, which may increase interest in girls to start using cannabis [19]. However, traditional gender norms can restrict girls’ access to cannabis [18], also resulting in less involvement in social networks of cannabis use, expressing concerns about control, but also not handling cannabis effects well [17]. Additionally, international research has indicated that the role of peers and friendships may operate differently for girls and boys [20]. This implies that the influence exerted by female peers on individuals may differ from the influence of male peers. However, to date, no study has specifically examined the differences in how girls and boys react to their same- or opposite-sex friends/peers regarding cannabis use. It highlights the importance of understanding the potentially distinct risk factors associated with social agents who could be found in their social environment (i.e., mother, father, sisters, brothers, female friends, male friends, female classmates, and male classmates for girls and boys.

Very few Spanish and Andalusian studies assessed potential differences between female and male adolescents about cannabis use. Romo-Avilés found a higher acceptance of cannabis use by girls [21]. Gonzalez-Amado, et al., examined the differences among young adults who cultivated cannabis regarding sociodemographic factors, patterns of use, and health problems, showing similar profiles between men and women [22]. Moreover, a recent qualitative study conducted in Andalusia explored the beliefs of regular cannabis users, but it did not specifically investigate the differences between girls and boys [23].

In response to the presented gaps, the objectives of this descriptive cross-sectional study are: first, to examine the prevalence rates of last month’s cannabis use by sex and consider the sociodemographic characteristics that are related to the use in girls and boys, and second, as the main goal of this study, to assess whether girls and boys would differ concerning certain beliefs about cannabis use (i.e., attitude, social influence, self-efficacy, coping plans, and intention). Specifically, a difference is done in the study of social environment influence in female influence VS male ‘influence (e.g., influence of female friends VS male friends). The findings of this study could be relevant to target cannabis prevention programs to the differences in beliefs between girls and boys, which could translate into a change in cannabis use behaviour.

Although the present study was conducted in western Andalusia, this represents the most densely populated region in southern Spain. The characteristics of this region could make the study not only representative of Spain but also other small countries such as Luxembourg, Belgium, or the Netherlands due to the vast extension of Andalusia.

## Methods

### Recruitment

**FIGURE 1:**
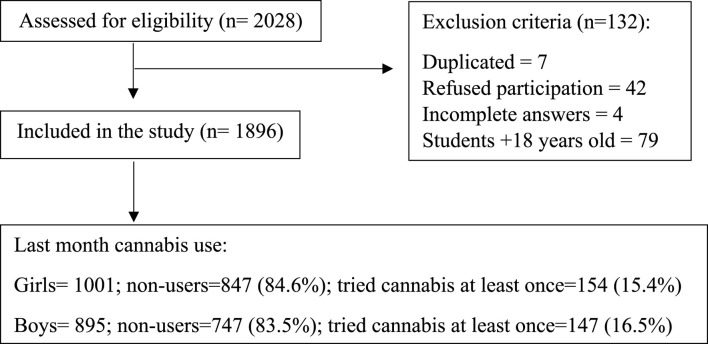
Flow diagram of the recruitment process based on the Strengthening the Reporting of Observational Studies in Epidemiology (STROBE) Statement (Bern, Switzerland, 2024).

Data were from participants aged 14–18 years from western Andalusia (Sevilla, Huelva, Córdoba, and Cádiz). High schools and classrooms were randomised selected. The online GRANMO tool was used to calculate the sample size (https://apisal.es/Investigacion/Recursos/granmo.html). It was estimated at 40% of dropout rate. First, we randomly selected at least two high schools from public centres and another two from private centres. In Spain, public schools are supported by the Public Government, and it is free to attend, while private schools are managed by a private entity (i.e., the admission process of the students is according to their criteria) and the financing is provided by parents. If a high school did not agree to participate, we randomly selected another high school in the same province. In total, we contacted 73 high schools and, finally, 21 high schools agreed to participate. However, one high school refused to participate once the study started (n = 20). A final sample of 2,028 students was recruited, of which 1,896 students were included in the current analysis (Figure 1).

Researchers determined eligibility based on inclusion criteria, which required adolescents enrolled in the third and fourth grade of compulsory secondary education (equivalent to the 9th and 10th grade in the United States), the first and second baccalaureate (equivalent to the 11th and 12th grade in the United States) and vocational training (VT). Participants older than 18 years old and duplicate or incomplete answers were excluded.

### Instrument

De Vries’ Integrated Change Model was used to design the survey [14], see for details supplementary material.

#### Sociodemographic Variables

Sex, age (in years), nationality (dichotomized into 1 = Spanish and 2 = other), having or not having religion, academic performance (0 = fail; 4 = excellent), adolescent’s educational level (1 = 9th grade, 2 = 10th grade, 3 = 11th grade, 4 = 12th grade and 5 = vocational training (VT); note that 11th and 12th grades prepare the student to access university studies, while vocational training has the purpose of teaching a trade), and parents’ educational level of parents (0 = none, 1 = primary studies, 2 = secondary education, 3 = university studies, and 8 = I do not have that relative), and having(1) or not having (0) a boyfriend/girlfriend. *Social status* was measured using the Family Affluence Scale (FAS III) [24], from the lowest purchasing power to the highest purchasing power (Cronbach’s α = 0.641). *Family functioning* was assessed using the Family Apgar test [25], consisting of five questions answered on a three-point Likert scale (0 = almost never, 1 = sometimes, 2 = almost always) (Cronbach’s α = 0.760). Finally, the adolescent received weekly pocket money (an amount of money).

#### Socio-Cognitive Variables

The socio-cognitive variables included five different constructs: attitude, social influence, self-efficacy, coping plans, and intention. Each construct is composed of a different number of items.

*Attitudinal beliefs (disadvantages and advantages)* of cannabis use were evaluated using a 5-point Likert scale to assess nine perceived pros (1 = strongly agree to 5 = strongly disagree; Cronbach’s α = 0.876) and nine cons (1 = strongly disagree to 5 = strongly agree; Cronbach’s α = 0.906) and assessed both rational consequences of using cannabis such us “cannabis causes family problems,” as emotional consequences (e.g., “it makes me feel guilty when I use it”).

*The social influence* scale measured three dimensions of social influence: social norms, social modelling, and social pressure. The influence of the same social agents was assessed for all three dimensions, resulting in a total of 11 social agents. These agents were differentiated by sex (female VS male), including mother, father, sister, brother, female friends, male friends, female best friends, male best friends, boyfriend/girlfriend, female classmates, and male classmates. This approach aimed to evaluate the role of social influence based on gender.

*Social norms* were assessed using the perceived opinion of the social environment on whether the respondent could consume cannabis or not (1 = definitely, it is okay to use cannabis; 5 = definitely, it is not okay to use cannabis; and 8 = I do not have that relative/friend) (Cronbach’s α = 0.916).

*Social modelling* with the same 11 agents as social norms (e.g., mother, father) assessed the frequency of cannabis consumption by individuals in the social environment of the respondent (1 = always; 5 = never, 8 = I do not have that relative/friend) (Cronbach’s α = 0.815).

*Social pressure*, with the same 11 agents as a social norm and social modelling, assessed to what extent the respondent has been under pressure to use cannabis by individuals in their social environment (1 = always; 5 = never, 8 = I do not have that relative/friend) (Cronbach’s α = 0.807).

Since not having that relative/friend does not influence behaviour, category 8 = I do not have that relative/friend, it was identified in the statistical software as a discrete missing value for a correct interpretation.

*Self-efficacy* assessed the perceived difficulty of adolescents to avoid cannabis use in 11 different situations, such as being at a party/celebration, feeling worried, or returning home after class (1 = strongly agree, which means low self-efficacy, to 5 = strongly disagree, which means high self-efficacy; Cronbach’s α = 0.986).

*Coping plans* were assessed by the same 11 items used in the self-efficacy scale, to assess which plans the respondent had made not to use cannabis in challenging situations (1 = strongly disagree, which means not having a coping plan toward cannabis use, to 5 = strongly agree, which means having a coping plan to refuse cannabis offer; Cronbach’s α = 0.992).

*The intention* of cannabis use in the next year and the future was assessed using a 7-point Likert scale (+3 = surely no; −3 = surely yes; Cronbach’s α = 0.867).

#### Cannabis Behaviour Variables

*Consumption of cannabis* was measured by asking the adolescent if the adolescent had ever tried cannabis in his life, and in the last month (never, 1 day, 2, 3–9, 10–19, or 20 days or more). The questions are from the Spanish ESTUDES survey [26].

### Procedure

Data collection was carried out by one researcher (MCTG) and took place in November-December 2020, after the lockdown in Spain. The lockdown took place in Spain from mid-March to the end of May. The return to school starting in June (corresponding to phase 2) was voluntary and with fewer students per classroom. After September 2020, normal school routine started to return, and schools agreed to evaluate participation in the study. Yet, school participation remained hindered, and prevailed in the execution of the intended 12-month follow-up.

Participants used schools’ computers or mobile phones to complete the online self-administered questionnaire which was previously validated by piloting [10] and lasted approximately 30 min of the class’s hour. An advantage of online surveys is that they do not allow the participant to advance without answering the first question. This ensures that there are no missing values. One of the researchers (MCTG) was present online or in person when high school allowed. The teachers of each class administered the link to the questionnaire when the center decided not to allow entrance at school or decided not to connect in an online meeting with the researcher.

All participants provided their written informed consent. Participants were instructed that they could choose not to complete the survey. Active written consent was requested from parents unless the high school indicated that they wanted to provide passive written consent (n = 7). Parents were notified in writing at least 1 week in advance of data collection, so they could opt out of their adolescent from data collection.

This observational study was approved by the Andalusian Research Ethics Committee (registration number: 0073-N-18) in 2018. The confidentiality of the data was guaranteed and explained to the participants and their parents, and the procedures followed the Regulation (EU) 2016/679 of the European Parliament and the Data Protection Council. In addition, during the design of the study, the data protection delegate of the University of Seville was contacted to carry out the corresponding procedures on the treatment of data of minors. This study was performed in line with the principles of the Declaration of Helsinki, and its later amendments or comparable ethical standards.

### Data Analysis

Descriptive statistics (mean and standard deviation for continuous variables and count and percentage for categorical variables) were used to describe the characteristics of the sample concerning sex. To investigate the association between sex and last month’s cannabis use, and sociodemographic variables, a *t*-test and chi-square test were performed depending on the type of variable. The reliability of scales was considered adequate if the Cronbach Alpha was at least 0.80.

For each construct of socio-cognitive (i.e., beliefs about attitude, 18 items; social influences, 33 items; self-efficacy, 11 items; coping plans, 11 items, and intention, 2 items), multivariate analyses of variance (MANOVA) were conducted to examine the differences between boys and girls, while the analysis was adjusted for last month cannabis use to exclude that the differences could have been caused by recent cannabis use. Values of 0.01, 0.06, and 0.14 can be considered as small, medium, and large effect sizes for MANOVA [27], though there is not a universally agreed-upon threshold for determining what constitutes a “small” or even “very small” effect size. The significance level was set at *p* ≤ 0.05. All the analyses were conducted in SPSS 28.0 version [28].

## Results

### Characteristics of the Sample and Cannabis Use According to Sex

Table 1 shows the characteristics of the overall sample and the differences between girls and boys. A little more than half of the participants were girls, from 10th grade, and public schools. The mean age of the participants was 15.48 years. Three-quarters of the students did not have a boyfriend/girlfriend, and most of them reported being Spanish and a little more than half of the participants did not have religion. Regarding differences between girls and boys and their sociodemographic characteristics, compared to boys, there were more girls in higher grades while there were more boys in vocational training, and girls had better academic performance than boys. In addition, boys reported having significantly more weekly pocket money than girls.

**TABLE 1:** Differences between girls and boys in sociodemographic variables and cannabis use behaviour (Andalucia, Spain, 2020).

Variables	Total participants	Girls				Boys N = 895 (47.2%)				Value of P from t^b^ and χ2^c^
	N^d^ = 1896	Total girls N = 1,001 (52.8%)	Non-users N = 847 (84.6%)	Users N = 154 (15.4%)	*p*-Value	Total boys N = 895 (47.2%)	Non-users N = 747 (83.5%)	Users N = 148 (16.5%)	*p*-Value	
Age (14–18) (mean, SD) (m^a^ 0)	15.48 (1.393)	15.45 (1.386)	15.38 (1.379)	15.82 (1.363)	**.000**	15.52 (1.401)	15.40 (1.424)	16.13 (1.093)	**.000**	.291
Adolescent Educational Level (m 0)									
• 9th grade	474 (25%)	248 (24.8%)	222 (26.2%)	26 (16.9%)	**.000**	226 (25.3%)	200 (26.8%)	26 (17.6%)	**.000**	**.000**
• 10th grade	581 (30.6%)	315 (31.5%)	269 (31.8%)	46 (29.9%)		266 (29.7%)	237 (31.7%)	29 (19.6%)		
• 11th grade	362 (19.1%)	196 (19.6%)	165 (19.5%)	31 (20.1%)		166 (18.5%)	137 (18.3%)	29 (19.6%)		
• 12th grade	354 (18.7%)	201 (20.1%)	165 (19.5%)	36 (23.4%)		153 (17.1%)	115 (15.4%)	38 (25.7%)		
• Vocational Training (VT)	125 (6.6%)	41 (4.1%)	26 (3.1%)	15 (9.7%)		84 (9.4%)	58 (7.8%)	26 (17.6%)		
Type of high school (m 0) • Public school • Private school	1,002 (53%) 888 (47%)	525 (52.5%) 475 (47.5%)	439 (51.8%) 408 (48.2%)	86 (55.8%) 67 (43.5%)	.334	477 (53.6%) 413 (46.4%)	395 (52.9%) 347 (46.5%)	82 (55.4%) 66 (44.6%)	.348	.334
Academic performance (mean, SD) (m 0)	2.63 (1.014)	2.75 (.988)	2.82 (.961)	2.36 (1.053)	**.000**	2.50 (1.027)	2.54 (1.002)	2.30 (1.128)	**.000**	**.000**
Mother educational level (m 0)									
• None • Basic or primary school• Secondary school• University studies• I do not have a mother	233 (12.3%) 355 (18.7%) 443 (23.4%) 627 (33.1%) 238 (12.6%)	133 (13.3%) 188 (18.8%) 228 (22.8%) 339 (33.9%) 113 (11.3%)	119 (14%) 160 (18.9%) 188 (22.2%) 285 (33.6%) 95 (11.2%)	14 (9.1%) 28 (18.2%) 40 (26%) 54 (35.1%) 18 (11.7%)	.499	100 (11.2%) 167 (18.7%) 215 (24%) 288 (32.2%) 125 (14%)	85 (11.4%) 137 (18.3%) 183 (24.5%) 244 (32.7%) 98 (13.1%)	15 (10.1%) 30 (20.3%) 32 (21.6%) 44 (29.7%) 27 (18.2%)	.479	.273
Father Educational Level (m 0)									
• None• Basic or primary school• Secondary school• University studies• I do not have a father	275 (14.5%) 407 (21.5%) 373 (19.7%) 550 (29%) 291 (15.3%)	160 (16%) 207 (20.7%) 191 (19.1%) 298 (29.8%) 145 (14.5%)	135 (15.9%) 178 (21%) 159 (18.8%) 249 (29.4%) 126 (14.9%)	25 (16.2%) 29 (18.8%) 32 (20.8%) 49 (31.8%) 19 (12.3%)	.841	115 (12.8%) 200 (22.3%) 182 (20.3%) 252 (28.2%) 146 (16.3%)	88 (11.8%) 168 (22.5%) 155 (20.7%) 216 (28.9%) 26 (16.1%)	27 (18.2%) 32 (21.6%) 27 (18.2%) 36 (24.3%) 26 (17.,6%)	.237	.227
Boyfriend/girlfriend (m 0)									
• No.• Yes	1,401 (73.9%) 495 (26.1%)	712 (71.1%) 289 (28.9%)	613 (72.4%) 234 (27.6%)	99 (64.3%) 55 (35.7%)	.053	689 (77%) 206 (23%)	603 (80.7%) 144 (19.3%)	86 (58.1%) 62 (41.9%)	**.000**	**.002**
Nationality (m 0)									
• Spanish• Non-Spanish	1855 (97.8%) 41 (2.2%)	981 (98%) 20 (2%)	830 (98%) 17 (2%)	151 (98.1%) 3 (1.9%)	1.00	874 (97.7%) 21 (2.3%)	731 (97.9%) 16 (2.1%)	143 (96.6%) 5 (3.4%)	.371	.358
Religion (m 0) • No • Yes	1,117 (58.9%) 779 (41.1%)	614 (61.3%) 387 (38.7%)	304 (35.9%) 543 (64.1%)	83 (53.9%) 71 (46.1%)	**.000**	503 (56.2%) 392 (43.8%)	304 (40.7%) 443 (59.3%)	88 (59.5%) 60 (40.5%)	**.000**	**.013**
Apgar test (m 3) • Severely dysfunctional family • Moderately dysfunctional family • Highly functional family	116 (6.1%) 327 (17.3%) 1,449 (76.6%)	68 (6.8%) 157 (15.7%) 773 (77.5%)	54 (6.4%) 131 (15.5%) 660 (77.9%)	14 (9.1%) 26 (16.9%) 113 (73.4%)	.380	48 (5.4%) 170 (19%) 676 (75.6%)	36 (4.8%) 143 (19.1%) 568 (76%)	12 (8.1%) 27 (18.2%) 108 (73%)	.259	.093
Family affluence (mean, SD) (m 1)	7.25 (2.702)	7.21 (2.702)	7.30 (2.692)	6.71 (2.712)	**.000**	7.30 (2.704)	7.33 (2.689)	7.14 (2.784)	**.000**	.479
Adolescent weekly pocket money (mean, SD) (m 0)	13.25 (14.277)	12.41 (12.007)	12.08 (12.193)	14.19 (10.793)	**.000**	14.20 (16.407)	12.87(14.22)	20.98 (23.621)	**.000**	**.006**

^a^ (m X): number of missing values per variable.

^b^ t: *t*-test.

^c^ χ2: Chi-square.

^d^ N: sample size.

Bold values represent significant values.

In this study, 302 (15.9%) students have tried cannabis in the last month, being more frequently used in boys. Female and male users were older, belonged to vocational training, had lower academic performance, reported more frequently not having a religion, and had lower family affluence (slightly higher difference in female users vs. female non-users). Moreover, the frequency of having a boyfriend or girlfriend was higher among users, but this was only statistically significant within the boys’ group, where users reported having a boyfriend or girlfriend twice as often as non-users. Finally, female and male users had more pocket money than female and male non-users; it is merely to note that male cannabis users reported to have €20.98, while female cannabis users reported to have €14.19.

### Socio-Cognitive Factors Toward Last Month’s Cannabis Use

Regarding attitudes, the boys are less convinced of all measured disadvantages of cannabis use than the girls. Yet, the girls are more convinced of some advantages of cannabis such as relieving boredom, using it as a medication, and stimulating creativity and imagination (see Table 2). Overall, the effect size was low. Medium effect sizes were found in 6 out of 9 items regarding the disadvantages of cannabis use while only one item showed a medium effect size for advantages of cannabis use (i.e., the medicinal use).

**TABLE 2:** Differences between girls and boys in attitudinal beliefs, social influence, self-efficacy, action plans, and intention concerning last month cannabis use (Andalucia, Spain, 2020).

	Girls		Boys		Difference	*p* ^a^	Effect size
	M^b^	SD^c^	M	SD			
Disadvantages^d^ (m^e^ 1) *Cannabis…*							
… It is bad for my health	4.47	0.868	4.25	1.070	F^f^ (1.17) = 20.60	**<.001**	.011
… gives me family problems	4.17	1.081	3.98	1.298	F (1.10) = 8.01	**.005**	.004
… gives me conflicts with peers	3.58	1.252	3.31	1.342	F (1.23) = 16.18	**<.001**	.009
… causes problems at school	4.00	1.192	3.76	1.355	F (1.16) = 11.90	**<.001**	.006
… causes memory problems	3.99	1.119	3.77	1.288	F (1.17) = 12.96	**<.001**	.007
… is addictive	4.51	.895	4.35	1.085	F (1.10) = 11.13	**<.001**	.006
… makes me feel ashamed of myself	3.37	1.343	3.16	1.404	F (1.14) = 8.65	**.003**	.005
… makes me feel guilty	3.76	1.230	3.52	1.355	F (1.15) = 10.57	**.001**	.006
… generates feelings of regret if I get sick after cannabis use	4.13	1.117	3.93	1.319	F (1.11) = 8.65	**.003**	.005
Advantages^g^ (m 0) *Cannabis…*							
… relaxes me	3.16	1.315	3.27	1.367	F (1.03) = 2.15	.142	.001
… makes me feel more secure with my friends	2.36	1.187	2.38	1.157	F (1.00) < 1	.828	.000
… makes me more popular	2.09	1.265	2.19	1.256	F (1.05) = 3.35	.067	.002
… makes me more sociable with others	2.27	1.274	2.28	1.259	F (1.00) < 1	.987	.000
… makes me feel more grown up	2.15	1.330	2.13	1.246	F (1.00) < 1	.813	.000
… relieves my boredom	2.24	1.263	2.40	1.306	F (1.08) = 5.95	**.015**	.003
… can be used as a medicine	3.31	1.313	3.56	1.293	F (1.24) = 15.05	**<.001**	.008
… stimulate my creativity and imagination	2.47	1.247	2.61	1.323	F (1.07) = 4.85	**.028**	.003
… helps me forget problems	2.78	1.388	2.70	1.378	F (1.05) = 2.97	.085	.002
Social norm^h^ (m 7) *Who thinks you can use cannabis?*							
My mother thinks …	4.92	.309	4.86	.388	F (1.01) = 12.62	**<.001**	.008
My father thinks …	4.90	.346	4.83	.504	F (1.02) = 11.86	**<.001**	.007
My sister (s) thinks (n) …	4.84	.471	4.79	.524	F (1.00) = 2.59	.108	.002
My brother (s) thinks (n) …	4.82	.502	4.75	.591	F (1.01) = 5.89	**.015**	.004
My Friends (female) think …	4.45	.801	4.38	.830	F (1.00) = 1.41	.235	.001
My friends (male) think …	4.36	.889	4.22	.946	F (1.07) = 6.90	**.009**	.004
My best friend female thinks …	4.67	.711	4.63	.761	F (1.00) < 1	.485	.000
My best friend male thinks …	4.69	.686	4.51	.868	F (1.12) = 18.89	**<.001**	.012
My boyfriend/girlfriend thinks …	4.86	.475	4.81	.584	F (1.00) = 2.60	.107	.002
My classmates (female) think…	4.42	.795	4.37	.809	F (1.00) < 1	.361	.001
My classmates (male) think…	4.33	.850	4.26	.900	F (1.01) = 1.55	.212	.001
Social Modelling^i^ (m 2) *Who of the people mentioned use cannabis … ?*							
My mother (or legal guardian)	4.96	.307	4.94	.362	F (1.00) = 1.36	.243	.001
My father (or legal guardian)	4.91	.449	4.89	.480	F (1.00) < 1	.477	.000
My sisters	4.96	.273	4.94	.346	F (1.00) < 1	.417	.000
My brothers	4.86	.566	4.88	.541	F (1.00) < 1	.355	.001
My Friends (female)	4.49	.921	4.53	.858	F (1.01) = 3.46	.063	.002
My Friends (male)	4.24	1.145	4.10	1.188	F (1.07) = 4.15	**.042**	.003
My best friend (female)	4.79	.676	4.85	.545	F (1.02) = 8.14	**.004**	.005
My best friend (male)	4.74	.766	4.69	.842	F (1.00) < 1	.662	.000
My boyfriend/girlfriend	4.86	.596	4.91	.482	F (1.00) = 4.02	**.045**	.003
My classmates (female)	4.46	.872	4.45	.923	F (1.00) < 1	.930	.000
My classmates (male)	4.27	.970	4.21	1.052	F (1.00) < 1	.318	.001
Social pressure^i^ (m 4) *I have felt pressured to use cannabis for…*							
My mother (or legal guardian)	4.98	.193	4.98	.197	F (1.00) < 1	.969	.000
My father (or legal guardian)	5.00	.137	4.98	.224	F (1.00) = 1.31	.234	.001
My sisters	5.00	.108	4.98	.219	F (1.00) = 1.53	.230	.001
My brothers	5.00	.084	4.99	.137	F (1.00) = 2.62	.137	.001
My Friends (female)	4.79	.578	4.90	.429	F (1.05) = 21.13	**<.001**	.013
My Friends (male)	4.77	.637	4.77	.590	F (1.00) < 1	.789	.000
My best friend (female)	4.92	.401	4.97	.218	F (1.01) = 12.33	**<.001**	.008
My best friend (male)	4.92	.412	4.92	.385	F (1.00) < 1	.775	.000
My boyfriend/girlfriend	4.98	.231	4.97	.232	F (1.00) < 1	.699	.000
My classmates (female)	4.91	.383	4.94	.313	F (1.01) = 3.31	**.030**	.003
My classmates (male)	4.89	.403	4.90	.397	F (1.00) < 1	.683	.000
Self-efficacy^g^ (m 2) *It is difficult NOT to use cannabis if…*							
My friends (female) offer me a joint	3.94	1.561	3.94	1.597	F (1.00) < 1	.973	.000
My friends (male) offer me a joint	3.91	1.572	3.87	1.581	F (1.01) < 1	.568	.000
I am worried	3.94	1.583	3.89	1.619	F (1.01) < 1	.519	.000
I feel great	3.95	1.582	3.93	1.620	F (1.00) < 1	.774	.000
I feel sad	3.88	1.587	3.89	1.594	F (1.00) < 1	.940	.000
I am stressed	3.88	1.580	3.86	1.592	F (1.00) < 1	.879	.000
I am at a party or celebration	3.74	1.568	3.76	1.599	F (1.00) < 1	.662	.000
I am in public spaces	3.98	1.585	3.97	1.598	F (1.00) < 1	.919	.000
I’m in others’ houses or mine when the parents are NOT	3.92	1.581	3.90	1.613	F (1.00) < 1	.870	.000
When returning home after class	4.06	1.594	4.02	1.627	F (1.00) < 1	.516	.000
I am alone	4.01	1.590	3.94	1.631	F (1.00) = 1.00	.353	.000
Coping plans^d^ (m 0) *I have made plans to make sure that I will not use cannabis…*							
My friends (female) offer me a joint	2.54	1.740	2.51	1.740	F (1.00) < 1	.939	.000
My friends (male) offer me a joint	2.55	1.732	2.54	1.731	F (1.00) < 1	.801	.000
I am worried	2.51	1.732	2.52	1.745	F (1.00) < 1	.768	.000
I feel great	2.53	1.767	2.51	1.760	F (1.00) < 1	.934	.000
I feel sad	2.51	1.728	2.52	1.756	F (1.00) < 1	.792	.000
I am stressed	2.51	1.716	2.54	1.741	F (1.00) < 1	.490	.000
I am at a party or celebration	2.54	1.675	2.56	1.706	F (1.00) < 1	.671	.000
I am in public spaces	2.56	1.774	2.51	1.759	F (1.00) < 1	.682	.000
I am in the homes of others or mine when the parents are NOT	2.54	1.744	2.55	1.765	F (1.00) < 1	.793	.000
When returning home after class	2.52	1.783	2.53	1.783	F (1.00) < 1	.788	.000
I am alone	2.52	1.769	2.52	1.772	F (1.00) < 1	.897	.000
Intention^j^ (m 2)							
Do you intend to use cannabis in the future?	1.11	1.223	1.03	1.403	F (1.00) < 1	.796	.000
Do you intend to use cannabis next year?	1.16	1.209	1.03	1.310	F (1.01) = 1.60	.206	.001

^a^ *p*: *p*-value.

^b^ M; mean.

^c^ SD: standard deviation.

^d^ Answer coding for disadvantages and coping plans: 1 = strongly disagree; 5 = strongly agree.

^e^(m X): number of missing values per scale.

^f^ F: ANOVA F.

^g^Answer coding for advantages and self-efficacy: 1 = Strongly agree; 5 = Strongly disagree.

^h^ Answer coding for social norms: 1 = I definitely can use cannabis; 5 = I definitely cannot use cannabis.

^i^ Answer coding for social modelling and pressure: 1 = always; 5 = never.

^j^ Answer coding for intention: Surely no = +3, Surely yes = −3.

Bold values represent significant values.

Regarding social norms, boys reported having more social norms favouring cannabis use than girls from their male social environment (i.e., norms favouring cannabis use from their father, brother, and male friends). Moreover, boys also reported having more norms favouring cannabis use from their mothers than girls. The effect size was nearly large for “best friend male” and medium for “parents.”

Regarding social modelling, the boys reported that their male friends use cannabis more often than the girls. Yet, the girls reported that their female best friends use cannabis more often than the boys. The same when referring to the boyfriend/girlfriend. The effect size was nearly medium for “female best friend.”

For social pressure, it was found that girls feel more pressure to use cannabis from their female friends, female best friends, and female classmates (i.e., female peers) compared to the boys. Female friends exert greater pressure to use cannabis than female best friends. The effect size is nearly large for “female friend” and medium for “female best friend.”

Girls and boys did not differ in self-efficacy either coping plans toward cannabis use or intention to use cannabis, (see Table 2).

## Discussion

First, this study aimed to investigate differences between girls and boys aged 14 to 18 in Andalusia regarding self-reported cannabis use in the last month, as well as sociodemographic factors associated with cannabis use. While cannabis consumption was significantly higher in boys, sociodemographic factors related to this were similar in both sexes, except for two factors: having boyfriend/girlfriend and pocket money. Having boyfriend or girlfriend was significant only in the boys’ group, where users reported having boyfriend/girlfriend twice as often as non-users. Pocket money among male users was considerably higher compared to the rest of the groups. Our results were similar to those of a recent study, which emphasized that cannabis consumers in early adolescence were predominantly identifying as male and had more weekly spending money [29]. For future studies, it is recommended to investigate the causality of these variables, as they may be determinants in cannabis consumption among males.

Second, there were examined the differences between girls and boys in certain beliefs about cannabis use. Regarding attitudinal beliefs, boys were less convinced of the disadvantages of cannabis use, most notably, concerning “cannabis use is bad for my health” and “it causes conflicts with peers.” For instance, one study demonstrated a greater societal perception that girls should not engage in risky behaviours [30] so this fact could be related to our results. Moreover, an international study, which showed similar results than ours, supported that boys have higher odds of positive attitudes towards the acceptability of cannabis use [31]. On the other hand, girls were more convinced of some advantages of cannabis use such as its medicinal use. Some studies indicated that women start using cannabis mainly for therapeutic purposes [19, 31], which may prove our findings.

In terms of social norms, our study found that boys are more significantly influenced by norms related to cannabis use, particularly by their parents, brothers, male friends and male best friend, compared to girls. This underscores the importance of targeting families, especially in the case of boys, as a pivotal group for cannabis prevention efforts [32]. By effectively guiding these families and establishing clear rules against cannabis use, there is potential for observing a positive impact and reduction in cannabis use, especially among males. Furthermore, in order to approach peer influence, it may be helpful to adopt a social network approach to analyze the specific number of friends and enhancing our ability to predict the impact of peers on cannabis use, as researcher could use these friendships as a preventive resource.

In terms of social modelling, consistent with the beliefs of our adolescent participants, male friends (including best friends) are perceived as more influent for boys than for girls, while, contrary, girls had greater influence by their female peers. This finding is inline with previous literature, and it may suggest that adolescents may be more influenced by peers of their own sex [33]. Moreover, in our study, girls showed increased social pressure compared to boys, especially from female peers (i.e., friends, best friend, and classmates) [34]. From a traditional perspective, it is plausible that boys may be less inclined to recognize social pressure, given societal expectations for them to maintain emotional resilience in public [17, 35]. Yet, another international study suggested boys feel more pressured to adopt risky behaviours, such as cannabis use [30]. Thus, more research is needed in order to draw clear conclusions and identifying if these influences could be determined by who is exerting influence (i.e., females or males). It must be noted that this is the first study to differentiate between the influences of female and male peers, so further research utilizing this design is warranted to provide a more comprehensive explanation and to bolster the robustness of our findings.

Neither self-efficacy nor coping plans were statistically significant for boys nor girls in the present study. This is understandable as most of the adolescents did not engage in cannabis use and thus may not yet encounter difficulties in resisting challenging situations. Yet, the means do show that they are not prepared for challenging situations as many boys and girls do not make any coping plans. A recent study comparing Andalucian cannabis users and non-users also showed that cannabis users had much lower self-efficacy than non-users [10]. Our results show that the scores of girls and boys on self-efficacy and coping plans do not differ and thus do not need a more targeted approach. Yet, international and national studies have shown the importance of self-efficacy and coping plans in the prevention of cannabis use in young people [36–38]. Thus, it would be recommended to focus the cannabis use prevention presentations on helping adolescents develop action skills and planning how to reject the offer of cannabis use (e.g., through role-play, and simulations), especially in boys, who are the ones who reported having the greatest intention to use in the future. Additionally, addressing the multiple contexts in which adolescents are embedded, such as our self-efficacy items, could help to target prevention programs.

Regarding the strengths of this study, one strong aspect of this study is the division of social agents into female and male categories. This segregation allows for a more detailed examination of the impact of social influence factors related to cannabis use in girls and boys. The study provides valuable insights into the different roles of the female social environment and male social environment in shaping beliefs regarding cannabis use. Moreover, this study holds significance as it is the first of its kind conducted in the adolescent population of Andalusia, examining beliefs and social influence related to cannabis use while differentiating between females and males. By focusing on the beliefs of these specific populations, the study contributes to a better understanding of the system of sex-gender in the context of cannabis use in Andalusian adolescents. For instance, this study facilitates the identification of the primary influencer’s social agents in cannabis consumption among adolescents in Andalusia. We could involve these influencers individuals to implement a peer-led intervention where they exert influence over the broader adolescent group, and that it will be sensitive to the differences between girls and boys that we found regarding some specific factors such as the disadvantages of cannabis use.

Regarding the limitations of this study, one is that the survey only included options for participants to identify themselves as either a boy or a girl. We did not include gender possibilities such as transgender or non-binary individuals. It is important to acknowledge that the sex-gender system is versatile, and while our study focused on sex as a biological characteristic, it can provide insights into gender associations, as the main focus is the beliefs. Additionally, as our study relied on cross-sectional data, it is not possible to determine the direction of the relationship between differences between girls and boys and beliefs about cannabis use. Longitudinal research would be needed to explore any causal or temporal relationships between these factors. Furthermore, it is worth noting that the effect size observed in our study was small. However, it is important to consider that small effect sizes are common in cannabis-related studies, yet they can still have significant implications [39]. These limitations provide opportunities for future research to expand on our findings and address the complexities of gender, incorporate diverse gender identities, employ longitudinal designs, and further explore the impact of socio-cognitive factors on beliefs about cannabis use. Finally, a last limitation is that the FAS scale in our study resulted in insufficient reliability, according to values considered as optimal by certain authors. However, other authors consider that values over 0.6 can be considered appropriated, and it is similar to the obtained in previous studies of validation of this scale [24, 40].

Regarding the implications for prevention field, to compare the difference in beliefs about cannabis use between girls and boys it is important to tailor interventions for cannabis use prevention. According to our findings, it is important to (1) address the detrimental effects of cannabis use, with particular emphasis on boys, (2) provide accurate and relevant information about attitudinal beliefs that are more pertinent to girls, such as the potential for freedom from boredom, medicinal use, and fostering creativity, and (3) increase both self-efficacy and coping plans in boys and girls, for instance, by developing refusal skills that enable adolescents to manage social influence, especially from peers (considering female peers for girls and males for boys). Strengthening social support systems for adolescents or equipping them with strategies to maintain and enhance high levels of self-efficacy to abstain from cannabis use is also recommended. This includes creating plans to reject invitations to use cannabis in general, as well as in specific high-risk situations and moods. By incorporating these recommendations, cannabis prevention programs can be tailored to address gender-specific factors and enhance their effectiveness in promoting healthy behaviours among adolescents. This gender-specific approach can enhance the effectiveness of prevention programs and ensure that interventions are relevant and resonant with the target audience.

To conclude, it is important to recognize the differences between girls and boys regarding beliefs related to cannabis use to tailor preventive interventions according to the unique challenges and vulnerabilities faced by girls and those which are faced by boys. The findings suggest that certain beliefs about cannabis use hold relatively more importance for girls compared to boys, and *vice versa*, so being sensitive to these differences may help to improve the effectives of the interventions. Prevention efforts should consider the potential barriers that adolescents face due to gender-related stigma, for instance, addressing the influence of male peers that promote cannabis use in boys, while interventions for girls may need to address female’ influence.
